# Additive prognostic value of longitudinal myocardial deformation to SCORE2 in psoriasis

**DOI:** 10.1093/ehjopen/oead016

**Published:** 2023-02-23

**Authors:** George Makavos, Ignatios Ikonomidis, Vaia Lambadiari, Georgia-Angeliki Koliou, George Pavlidis, John Thymis, Pinelopi Rafouli-Stergiou, Gavriella Kostelli, Konstantinos Katogiannis, Konstantinos Stamoulis, Aikaterini Kountouri, Emmanouil Korakas, Kostas Theodoropoulos, Alexandra Frogoudaki, Pelagia Katsimbri, Evangelia Papadavid

**Affiliations:** 2nd Department of Cardiology, Attikon Hospital, Medical School, National and Kapodistrian University of Athens, Rimini 1, Haidari, Athens 12462, Greece; 2nd Department of Cardiology, Attikon Hospital, Medical School, National and Kapodistrian University of Athens, Rimini 1, Haidari, Athens 12462, Greece; 2nd Department of Internal Medicine Propaedeutic, Research Institute and Diabetes Center, Attikon Hospital, Medical School, National and Kapodistrian University of Athens, Rimini 1, Haidari, Athens 12462, Greece; 2nd Department of Cardiology, Attikon Hospital, Medical School, National and Kapodistrian University of Athens, Rimini 1, Haidari, Athens 12462, Greece; 2nd Department of Cardiology, Attikon Hospital, Medical School, National and Kapodistrian University of Athens, Rimini 1, Haidari, Athens 12462, Greece; 2nd Department of Cardiology, Attikon Hospital, Medical School, National and Kapodistrian University of Athens, Rimini 1, Haidari, Athens 12462, Greece; 2nd Department of Cardiology, Attikon Hospital, Medical School, National and Kapodistrian University of Athens, Rimini 1, Haidari, Athens 12462, Greece; 2nd Department of Cardiology, Attikon Hospital, Medical School, National and Kapodistrian University of Athens, Rimini 1, Haidari, Athens 12462, Greece; 2nd Department of Cardiology, Attikon Hospital, Medical School, National and Kapodistrian University of Athens, Rimini 1, Haidari, Athens 12462, Greece; 2nd Department of Cardiology, Attikon Hospital, Medical School, National and Kapodistrian University of Athens, Rimini 1, Haidari, Athens 12462, Greece; 4th Department of Internal Medicine, Attikon Hospital, Medical School, National and Kapodistrian University of Athens, Rimini 1, Haidari, Athens 12462, Greece; 4th Department of Internal Medicine, Attikon Hospital, Medical School, National and Kapodistrian University of Athens, Rimini 1, Haidari, Athens 12462, Greece; 2nd Department of Dermatology and Venereology, Attikon Hospital, Medical School, National and Kapodistrian University of Athens, Rimini 1, Haidari, Athens 12462, Greece; 2nd Department of Cardiology, Attikon Hospital, Medical School, National and Kapodistrian University of Athens, Rimini 1, Haidari, Athens 12462, Greece; 4th Department of Internal Medicine, Attikon Hospital, Medical School, National and Kapodistrian University of Athens, Rimini 1, Haidari, Athens 12462, Greece; 2nd Department of Dermatology and Venereology, Attikon Hospital, Medical School, National and Kapodistrian University of Athens, Rimini 1, Haidari, Athens 12462, Greece

**Keywords:** Global longitudinal strain, Psoriasis, Prognosis, Pulse wave velocity, Biological agents

## Abstract

**Aims:**

Psoriasis has been associated with increased cardiovascular (CV) risk. We investigated whether markers of CV function and their change after treatment have a prognostic value for adverse outcomes.

**Methods and results:**

In a prospective study, at baseline and after 6 months of treatment with biological agents, we assessed in 298 psoriasis patients (i) left ventricular global longitudinal strain (GLS) and (ii) carotid-femoral pulse wave velocity (PWV), to evaluate their prognostic value for major adverse cardiovascular events (MACEs), including coronary artery disease, stroke, hospitalization for heart failure, and all-cause death over a 4-year follow-up period. During follow-up, 26 (8.7%) MACEs were recorded. By univariate analysis, decreasing absolute GLS values [hazard ratio (HR): 0.73, *P* < 0.001], decreasing GLS change after treatment (HR: 0.53, *P* = 0.008), and increasing PWV values (HR: 1.16, *P* = 0.049) were associated with adverse outcomes. Baseline GLS and its change post-treatment remained independent predictors of adverse events after adjusting for several confounders (*P* < 0.05). The addition of baseline GLS and its absolute change post-treatment to SCORE2 increased Harrell’s *C* from 0.882 to 0.941. By multivariable analysis, for each 1% increase in absolute baseline GLS values, the risk of MACE decreased by 33% and for each 1% absolute increase of GLS post-treatment compared with the baseline value, the risk of MACE decreased by 58%.

**Conclusion:**

Global longitudinal strain has an independent and additive prognostic value to SCORE2 for adverse CV events in psoriasis, providing timely decision-making for intensive anti-inflammatory treatment and aggressive modification of risk factors to reduce CV risk.

## Graphical Abstract

**Graphical Abstract:**
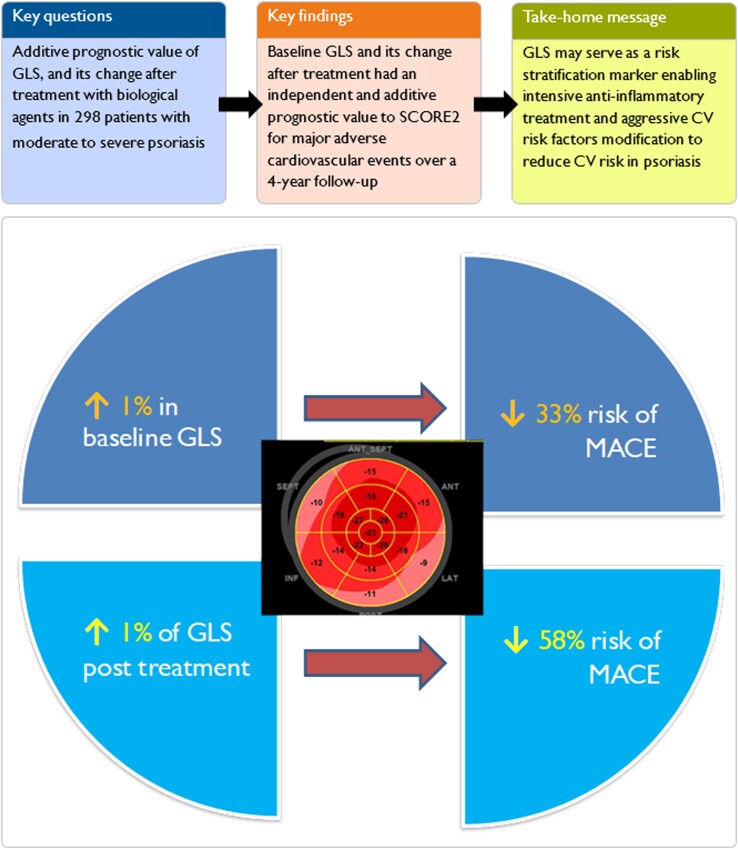

## Introduction

Psoriasis is a common, immune-mediated disease affecting up to 3% of the general population, characterized by skin lesions and chronicity. Systemic manifestations may also occur, associated with accelerated atherosclerosis and increased cardiovascular (CV) risk.^1-3^

Atherosclerosis and psoriasis share common pathophysiological pathways including inflammation, oxidative stress, and common genetic susceptibility.^4^ Global longitudinal strain (GLS) and pulse wave velocity (PWV) are both similarly impaired in psoriasis and coronary artery disease (CAD) patients,^5^ findings consistent with subtle myocardial and vascular dysfunction, possibly attributed to a similarly increased inflammatory and oxidative stress burden.^5^

Psoriasis has been proposed as an independent risk factor for adverse CV events.^3,6-9^ Epidemiological studies have demonstrated higher rates of established CV risk factors in psoriasis compared with the general population,^10-15^ and current recommendations suggest CV risk estimation according to CV disease risk prediction models.^16^

However, the prognostic value of markers of subclinical myocardial and vascular dysfunction for adverse CV events has not been adequately studied in psoriasis. We aimed to evaluate the prognostic value of markers of myocardial function, namely GLS (assessed by two-dimensional speckle-tracking echocardiography), and arterial stiffness (assessed by PWV) in psoriatic patients at baseline and after anti-inflammatory treatment with biological agents and whether these markers may provide additive prognostic value over traditional CV risk factors and the recently updated SCORE2^16^ for CV events.

## Methods

### Study population

We prospectively enrolled 298 patients (from September 2014 until December 2017) with moderate-to-severe psoriasis. Baseline clinical characteristics of the study population are presented in *Table 1*. All patients had received treatment with biological agents (either anti-TNF-α or anti-interleukin-12/23 or anti-interleukin-17 inhibitors) in the 2nd Department of Dermatology and Venereology, Attikon Hospital, University of Athens, for at least 6 months regardless of the discontinuation or combined use of other oral agents or phototherapy during the follow-up period. Baseline assessment date was defined as the initiation date of treatment with biological agents. One hundred and thirty-eight patients were treated with anti-TNF-α agents, 89 patients with anti-interleukin 12/23, and 71 patients with anti-interleukin 17a agents.

**Table 1:** Baseline clinical characteristics of the study population

	All patients (*N* = 298)	Patients without MACE (*N* = 272)	Patients with MACE (*N* = 26)	*P*-value
Age, year	51.6 ± 12.7	50.5 ± 12.6	62.5 ± 5.6	**<0.001**
Sex (male), *n* (%)	179 (60.1)	161 (59.2)	18 (69.2)	0.318
PASI score	12.30 ± 3.6	12.30 ± 3.6	11.90 ± 3.8	0.905
Disease duration (years)	17 ± 12	17.3 ± 12	12.3 ± 9	0.116
Risk factors, *n* (%)				
Hypertension	100 (33.6)	80 (29.4)	20 (76.9)	**<0**.**001**
Hyperlipidaemia	98 (32.9)	82 (30.1)	16 (61.5)	**0**.**001**
Diabetes	53 (17.8)	34 (12.5)	19 (73.1)	**<0**.**001**
Current smoking	142 (47.7)	130 (47.8)	12 (46.2)	0.846
SCORE2 (median; IQR)	2 (0–9)	1 (0–6)	10 (9–12)	**0**.**001**
Medications, *n* (%)				
ACE inhibitors/ARBs	91 (30.4)	73 (26.8)	18 (69.2)	**<0**.**001**
Diuretics	20 (6.7)	17 (6.3)	3 (11.5)	0.303
Lipid-lowering drugs	96 (32.2)	84 (30.9)	12 (46.2)	0.111
SBP (mmHg)	135.2 ± 20.6	134.2 ± 20	145.6 ± 21	**0**.**009**
DBP (mmHg)	82.6 ± 12.3	82.2 ± 12.5	86.7 ± 9.3	**0**.**040**
hs-CRP (mg/L)	5.9 ± 4.5	5.8 ± 4.9	6 ± 2.8	0.940
HbA1c (%)	6.7 ± 0.8	6.6 ± 0.7	6.8 ± 1	0.818
Total cholesterol (mg/dL)	193.5 ± 42.9	194.3 ± 42.8	186.6 ± 45	0.514
LDL-C (mg/dL)	126.4 ± 36.5	127.4 ± 35.8	117.2 ± 43.2	0.309
HDL-C (mg/dL)	47.8 ± 13	48 ± 13.4	46.4 ± 7.5	0.474
Triglycerides (mg/dL)	139 ± 74.2	137.2 ± 74.8	154.6 ± 68.6	0.391

Data are presented as mean values ± standard deviation and number (%). SCORE2 as median and interquartile range (IQR). All patients were treated with biological agents. Bold values indicate statistically significant differences between values (*P* < 0.05).

PASI, psoriasis area severity index; ACEis, angiotensin-converting enzyme inhibitors; ARBs, angiotensin II receptor blockers; SBP, systolic blood pressure; DBP, diastolic blood pressure; hs-CRP, high-sensitivity C-reactive protein; HbA1c, glycosylated haemoglobin; LDL-C, low-density lipoprotein cholesterol; HDL-C, high-density lipoprotein cholesterol.

At baseline and after 6 months of treatment with biological agents (within 1 week after completion of the 6-month period), we assessed (i) left ventricular ejection fraction (LVEF), (ii) GLS, and (iii) PWV. We also assessed left ventricular (LV) diastolic function parameters, namely peak mitral inflow velocity (*E*), deceleration time (DT) of *E*-wave isovolumic relaxation time (IVRT), early diastolic mitral annulus velocity (*E*ʹ) by tissue Doppler imaging, *E*/*E*ʹ, Left atrial (LA) volume index, as well as right ventricular (RV) function parameters, namely systolic tricuspid annulus velocity (SʹRV), by tissue Doppler imaging. The mean follow-up period was 4 years (48 ± 2 months) for incidence of major adverse cardiovascular events (MACEs) defined as the composite endpoint of one of the following: (i) CAD including angina pectoris and acute coronary syndrome, (ii) stroke, (iii) hospitalization for heart failure, and (iv) all-cause mortality. The patients were observed from the 1st day after the completion of the 6-month treatment with biological agents, until the occurrence of their 1st MACE or until they reached the end of the follow-up period without MACE. Cardiovascular risk factors included smoking, hypertension, hyperlipidaemia, and diabetes mellitus. The recently updated SCORE2 for CV events based on age, sex, blood pressure, smoking, and non-HDL cholesterol was also calculated in all patients.

Baseline exclusion criteria were history of CAD diagnosed before baseline assessment, presence of LV wall motion abnormalities and ejection fraction of <50%, severe valvular heart disease, primary cardiomyopathies, malignancies, psoriatic arthritis as it represents a different disease entity and treatment with b-blockers, as b-blockers may induce or exacerbate psoriasis. Coronary artery disease with significant epicardial stenosis (>70%) at baseline was excluded in psoriatic patients by absence of clinical history, angina, and reversible myocardial ischaemia, as assessed by treadmill test and stress echocardiography or computed tomography coronary angiography. The disease duration from initial diagnosis until inclusion in the study was 17 ± 12 years. After the exclusion of 28 patients because of inadequate speckle-tracking echocardiography images for analysis (91.4% feasibility), the final cohort included in the study was 298 patients. Echocardiographic and vascular function markers are listed in *Table 2*.

**Table 2:** Echocardiographic and vascular function markers of the study population (*N* = 298)

	All (*Ν* = 298)			Without MACE (*Ν* = 272)			With MACE (*Ν* = 26)		
	Baseline	After treatment	*P*-value	Baseline	After treatment	*P*-value	Baseline	After treatment	*P*-value
E (cm/s)	72.7 ± 16.8	71.6 ± 16.2	0.311	72.7 ± 16.6	71.7 ± 15.4	0.312	72.8 ± 19.2	71.2 ± 22.3	0.508
Eʹ (cm/s)	11.3 ± 3.6	11.3 ± 3.7	0.723	11.6 ± 3.6	11.7 ± 3.7	0.821	8.7 ± 1.7	9 ± 2	0.511
E/Eʹ	6.91 ± 2.7	6.81 ± 2.4	0.621	6.66 ± 2.5	6.57 ± 2.2	0.743	8.83 ± 3.8	8.62 ± 3.4	0.623
Sʹ (cm/s)	9.6 ± 4.1	9.9 ± 6.8	0.600	9.75 ± 4.2	10.1 ± 7.3	0.644	8.7 ± 3.1	8.4 ± 2	0.732
RV Sʹ (cm/s)	13.1 ± 2.7	13.2 ± 3.3	0.601	13.2 ± 2.7	13.4 ± 3.3	0.501	12.1 ± 2	11.8 ± 2.7	0.667
DT (ms)	209.4 ± 51.7	209.3 ± 57.5	0.911	206.3 ± 51.3	207.6 ± 56	0.811	235.6 ± 49.1	223.5 ± 69.4	0.402
IVRT (ms)	88.9 ± 22.2	88.5 ± 24.1	0.812	89.1 ± 22.8	87.8 ± 23.7	0.546	87.2 ± 17.1	94.6 ± 27	0.05
LA volume index (mL/m^2^)	24.8 ± 10.3	25.2 ± 10.7	0.331	24.1 ± 10.3	24.4 ± 10.7	0.412	30.5 ± 8.9	31.7 ± 8.6	0.308
LVEF (%)	57.6 ± 4.6	58.1 ± 4.4	0.721	58.1 ± 4.5	58.5 ± 4.4	0.644	57.5 ± 4.4	57.9 ± 4.5	0.711
GLS (%)	17.58 ± 2.5	18.72 ± 2.1	<0.001	17.8 ± 2.4	19.1 ± 1.9	<0.001	15.21 ± 1.9	15.72 ± 1.8	0.060
PWV (m/s)	10.75 ± 2.5	10.20 ± 1.8	<0.001	10.67 ± 2.5	10.13 ± 1.8	<0.001	11.50 ± 2.5	11.02 ± 1.7	0.051

E, peak mitral inflow wave; DT, deceleration time of mitral inflow E wave; IVRT, isovolumic relaxation time; LA, left atrial; Sʹ, systolic mitral annulus velocity by tissue Doppler imaging (average of lateral and septal velocities); Eʹ, diastolic mitral annulus velocity by tissue Doppler imaging (average of lateral and septal velocities); RV Sʹ, systolic tricuspid annulus velocity by tissue Doppler imaging; LVEF, left ventricular ejection fraction; GLS, global longitudinal strain; PWV, pulse wave velocity.

The psoriasis area severity index (PASI) was used to monitor the extent of disease and was calculated at baseline and after a 6-month treatment with biological agents to monitor the effect of treatment. In all patients, LV function and vascular function assessment were performed on the same day. The study protocol was approved by the Institute’s Ethics Committee, and written informed consent was obtained from all patients.

### Arterial stiffness

Carotid-femoral PWV was measured using a previously published methodology (Complior; Alam Medical, Vincennes, France).^5^ Pulse wave velocity was calculated as the distance divided by transit time between waves (m/s).

### Echocardiography

#### Two-dimensional and speckle-tracking echocardiography

Studies were performed in the echocardiography laboratory of the 2nd Department of Cardiology, Attikon Hospital, University of Athens, using a Vivid E95 (GE Medical Systems, Horten, Norway) ultrasound system. All studies were digitally stored in a computerized station (Echopac 204; GE Medical Systems, Horten, Norway) and were analysed by two observers, blinded to clinical and laboratory data. From cross-sectional echocardiographic images, we measured LV end-diastolic and end-systolic diameter (mm), interventricular septal and posterior wall thickness (mm), LV end-diastolic volume (mL), LV end-systolic volume (mL), and ejection fraction (%) using Simpson method of discs. Left atrial volume (mL) was measured from four- and two-chamber views, using the disk summation method and indexed to body surface area as LA volume index (mL/m^2^).

Using a dedicated software package (EchoPAC), two-dimensional strain was measured using speckle-tracking analysis. We acquired LV apical four-, two-, and three-chamber views at frame rates ≥50 frames/s. Subsequently, we calculated the GLS from the apical views (four, two, and three chambers) according to previously published methodology.^17^ All variables represent the mean value of measurements taken in three consecutive cardiac cycles. Patients with ≥2 segments with poor image quality were rejected from the analysis. The inter- and intra-observer variabilities of GLS were 10 and 7%, respectively. The change of GLS post-treatment was calculated as baseline GLS values minus GLS value at 6 months. For purposes of clarity in presentation of results, the GLS values are presented as absolute positive values.

#### Doppler echocardiography

The early mitral inflow *E* wave was measured by using pulsed-wave Doppler. The DT of *E* mitral wave and the IVRT between aortic closure and beginning of mitral *E* wave were measured in Doppler mitral inflow recordings. Myocardial velocities were recorded with tissue Doppler imaging. The sample volume was placed in the septal and lateral sites of the mitral annulus in the apical four-chamber view to record the LV systolic velocity (*S*ʹ) and early diastolic velocity (*E*ʹ). The average value of the velocities at the two annular sites was used to calculate *S*ʹ and *E*ʹ. The ratio of the mitral *E*, the *E*ʹ (*E*/*E*ʹ), was also calculated. The sample volume was also placed in the anterior site of the tricuspid annulus to record the RV systolic velocity (RV *S*ʹ).

### Statistical analysis

Continuous variables were tested for normality using the Kolmogorov–Smirnov test. Normally distributed variables were expressed as mean ± standard deviation (SD). Hazard ratios (HRs) with the respective 95% confidence intervals (CIs) were obtained through univariate and multivariable Cox regression analyses to estimate the risk of MACE for the examined markers of myocardial and vascular function and their change after treatment, age, sex, PASI, CV risk factors, and medication. In multivariable analysis, three different models were examined using both forward and backward selection procedure with the following parameters included in Model 1: SCORE2, diabetes, PASI, LVEF, PWV, treatment with angiotensin-converting enzyme inhibitors (ACEis)/angiotensin II receptor blockers (ARBs), lipid-lowering drugs, Model 2: all parameters of Model 1 plus GLS baseline to assess its additive predictive value and Model 3: all parameters of Model 2 plus GLS absolute change post-treatment. This analysis was applied for each one of the examined echocardiography markers.

Harrell’s *C* statistic was calculated to evaluate the improvement in risk prediction.

Survival curves were estimated using the Kaplan–Meier method for GLS (using as cut-off the lowest tertile of GLS) and for the absolute GLS difference (according to the tertiles of the change of GLS post-treatment). The log-rank test for time-to-event data with respect to the total events was used for comparison among groups. We planned a study with an accrual interval of 1 year, and additional follow-up after the accrual interval of 4 years. Based on our previous studies, we speculated that the median survival time of patients with normal GLS (greater than absolute value 17% median of the overall population) would be 5 years. If the true HR (relative risk) of patients with normal GLS relative to those with impaired GLS is 2, we estimated that we would need to study 128 patients with impaired and 128 patients with normal GLS to be able to reject the null hypothesis that the normal and abnormal GLS survival curves are equal with probability (power) 0.850. The Type I error probability associated with this test of this null hypothesis is 0.05.

Statistical analysis was conducted using SPSS (version 26; SPSS, Chicago, IL, USA), Stata (version 16 Stata Corp LP, College Station, TX, USA), and SAS (version 9.3, SAS Institute, Inc., Cary, NC, USA). All tests were two-sided. Significance was set at 5%.

## Results

We prospectively enrolled 298 patients (51.6 ± 12.7 years, 179 men) with PASI disease activity score: 12.3 ± 3.6 SD. During the 4-year follow-up period, 26 MACEs occurred (8.72% of the study population). Coronary artery disease occurred in eight patients (2.68%), stroke in eight patients (2.68%), hospitalization for heart failure in eight patients (2.68%), and all-cause death in two patients (0.67%). At the end of treatment with biological agents, all patients had improved GLS (from −17.58 ± 2.5 at baseline to −18.72 ± 2.1 after treatment, *P* < 0.001) and PWV (from 10.75 ± 2.5 at baseline to 10.20 ± 1.8 after treatment, *P* < 0.001) values (*Table 2*). No significant effect was observed after treatment in mitral *E*, IVRT, DT, *E*ʹ, *E*/*E*ʹ, RV *S*ʹ, and LA volume index (*P* > 0.05). In patients free of MACE, there was a greater improvement of GLS compared with those with MACE (GLS increase 1.30 vs. 0.51%, respectively, *P* = 0.001). Lower PWV values were observed after 6 months of treatment in patients without MACE (from 10.67 ± 2.5 at baseline to 10.13 ± 1.8 after treatment, *P* < 0.001) but not in patients with MACE (from 11.50 ± 2.5 at baseline to 11.02 ± 1.7 after treatment *P* = 0.050) (*Table 2*). Psoriasis area severity index decreased from 12.30 ± 3.6 at baseline to 2.30 ± 2 at 6 months of treatment (*P* < 0.001) in all patients. Psoriasis area severity index was similarly decreased in patients with (from 11.90 ± 3.8 to 2.54 ± 2 *P* < 0.001) or without MACE (from 12.30 ± 3.6 to 2.30 ± 2, *P* < 0.001). Baseline GLS and GLS change correlated modestly with disease duration (*r* = −0.17, *P* = 0.012 and *r* = 0.23, *P* = 0.001, respectively). By univariate Cox regression analysis, age and the presence of hypertension, diabetes mellitus, and hyperlipidaemia were associated with incidence of MACE (*P* < 0.05 for all associations) (*Table 3*). SCORE2 was also a univariate predictor of MACE (HR: 1.27, 95% CI: 1.18–1.36, *P* < 0.001). There was no association between disease duration and incidence of MACE (*P* = 0.2). Among the markers of myocardial and vascular function, decreasing absolute baseline values of GLS (HR: 0.73, 95% CI: 0.65–0.83, *P* < 0.001) and lower values of GLS absolute change after treatment (HR: 0.53, 95% CI: 0.33–0.84, *P* = 0.008) were associated with incidence of MACE (*Table 3*). No association was found between decreasing LVEF with MACE incidence (*P* = 0.15). Although increasing PWV values had a borderline predictive value for MACE (HR: 1.16, 95% CI: 1.00–1.33, *P* = 0.049) (*Table 3*), the change of PWV after treatment did not show a significant association with MACE (*P* = 0.820). By multivariable analysis, using the backward procedure, SCORE2, decreasing baseline GLS and lower GLS absolute change after treatment remained independent predictors of increased risk for MACE (*P* < 0.05) (*Table 4*). For each 1% increase in absolute baseline GLS values, the risk of MACE decreased by 33%, and for each 1% absolute increase of GLS change post-treatment compared with the baseline value, the risk of MACE decreased by 58%. (multivariable Model 3).

**Table 3:** Risk of MACE according to age, sex, CV risk factors, echocardiographic markers of vascular function and medication

Univariate analysis		
Covariates	Hazard ratio (95% CI)	*P*-value
Age	**1.08** (**1.05–1.12)**	**<0**.**001**
Sex (male)	1.32 (0.57–3.09)	0.518
PASI	0.98 (0.92–1.04)	0.459
Hyperlipidaemia	**2.50** (**1.06–5.88)**	**0**.**036**
Diabetes mellitus	**11.73**(**4.90–28.11)**	**<0**.**001**
Hypertension	**5.24** (**2.08–13.20)**	**<0**.**001**
SCORE2	**1.27** (**1.18–1.36)**	**<0**.**001**
Disease duration	0.97 (0.92–1.02)	0.211
Current smoking	0.73 (0.34–1.57)	0.420
E	2.28 (0.15–34.59)	0.554
Eʹ	**0.08** (**0.01–0.63)**	**0**.**016**
E/Eʹ	**1.21** (**1.09–1.35)**	**0**.**001**
Sʹ	0.46 (0.17–1.29)	0.140
RV Sʹ	0.60 (0.18–2.06)	0.420
DT	**1.01** (**1.00–1.02)**	**0**.**023**
IVRT	0.99 (0.97–1.02)	0.672
LA volume index	**1.04** (**1.01–1.07)**	**0**.**013**
LVEF	0.94 (0.86–1.02)	0.150
PWV	**1.16** (**1.00–1.33)**	**0**.**049**
PWV change	0.95 (0.63–1.45)	0.820
GLS baseline	**0.73** (**0.65–0.83)**	**<0**.**001**
GLS absolute change	**0.53** (**0.33–0.84)**	**0**.**008**
ACEis/ARBs	0.98 (0.18–5.34)	0.980
Lipid-lowering drugs	0.35 (0.10–1.24)	0.111

Bold values indicate statistically significant values (*P* < 0.05). PASI, psoriasis area severity index; ACEis, angiotensin-converting enzyme inhibitors; ARBs, angiotensin II receptor blockers; LVEF, left ventricular ejection fraction; GLS, global longitudinal strain; PWV, pulse wave velocity; E, peak mitral inflow wave; DT, deceleration time of mitral inflow E wave; IVRT, isovolumic relaxation time; LA, left atrial; Sʹ, systolic mitral annular velocity by tissue Doppler imaging (average of lateral and septal velocities); Eʹ, diastolic mitral annulus velocity by tissue Doppler imaging (average of lateral and septal velocities); RV Sʹ, systolic tricuspid annulus velocity by tissue Doppler imaging; MACE, major adverse cardiovascular events.

**Table 4:** Additive predictive value of myocardial deformation markers to SCORE2

Multivariable models		
Parameter	HR (95%CI)	*P*-value
Model 1		
SCORE2	1.27 (1.19–1.37)	<0.001
Diabetes	3.77 (1.36–10.42)	0.011
Harrell’s C	0.882	
Model 2		
SCORE2	1.25 (1.14–1.37)	<0.001
GLS baseline	0.78 (0.68–0.90)	0.003
Harrell’s C	0.913	
Model 3		
SCORE2	1.21 (1.08–1.35)	<0.001
GLS baseline	0.67 (0.58–0.78)	<0.001
GLS absolute change	0.42 (0.25–0.71)	0.006
Harrell’s C	0.941	

Model 1: The following parameters included in the initial step of multivariable analysis: SCORE2, diabetes, PASI, LVEF, PWV, treatment with angiotensin-converting enzyme inhibitors (ACEis)/angiotensin II receptor blockers (ARBs), and lipid-lowering drugs. The last step of the backward procedure in Cox regression analysis is shown. HR (95%CI), hazard ratio and 95% confidence intervals.

Model 2: Model 1 plus GLS baseline.

Model 3: Model 2 plus GLS absolute change post-treatment.

The addition of baseline GLS to SCORE2 increased Harrell’s *C* from 0.882 to 0.913 (*P* < 0.001), and in a next step, the addition of GLS absolute change post-treatment to SCORE2 and baseline GLS further increased Harrell’s *C* to 0.941 (*P* < 0.001).

The HR and significance of baseline GLS and its absolute increase after treatment remained similar after addition of disease duration (*P* > 0.05; data not given). There was no difference in terms of MACE incidence between the type of biological treatment (either anti-TNF-α or anti-IL-12/23 or anti-IL-17a) after adjustment for age, sex, PASI, CV risk factors, treatment with ACEis/ARBs, lipid-lowering drugs, and vascular and myocardial function markers (*P* > 0.05; data not available).

**Figure 1:**
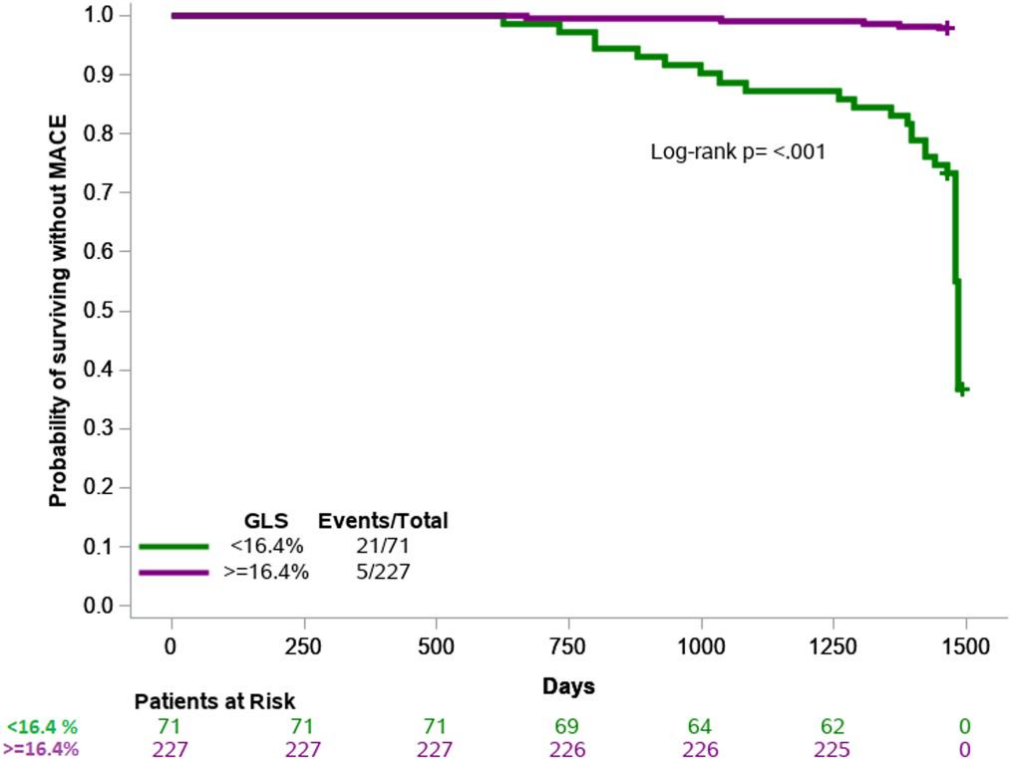
Kaplan–Meier curve for global longitudinal strain (using the −16.4% as a cut-off value) with respect to survival without a major adverse cardiovascular event.

We also conducted Kaplan–Meier survival time analysis for participants with absolute baseline GLS ≥ 16.4% and those with baseline GLS <16.4% (value of the lowest tertile) (*Figure 1*). We observed a lower-cumulative MACE-free survival in patients with baseline GLS < 16.4% compared with the group with baseline GLS ≥ 16.4% (log rank *P* = <0.001). In detail, only 5 of 226 patients with baseline GLS ≥ 16.4 had a MACE (absolute risk 2.2%), whereas 21 of 72 with a baseline GLS < 16.4% had a MACE (absolute risk 29.2%).

**Figure 2:**
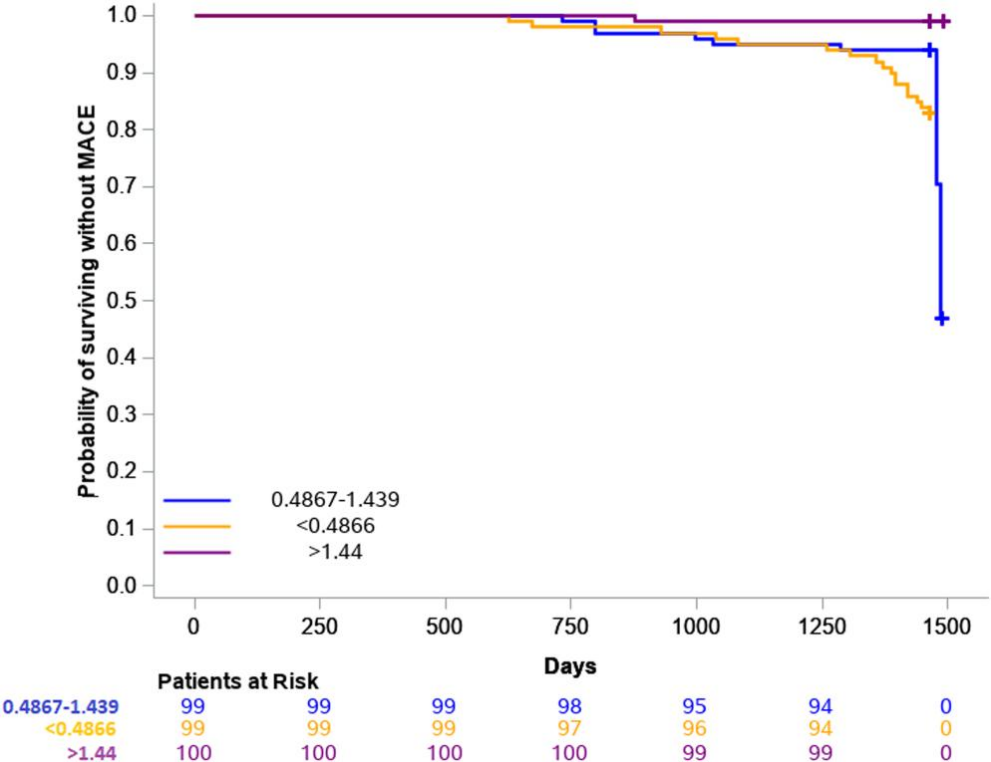
Kaplan–Meier curve for global longitudinal strain change with respect to survival without a major adverse cardiovascular event.

By Kaplan–Meier analysis, we also analysed the tertiles of the GLS absolute increase after 6 months of treatment. Participants with a GLS absolute increase of ≥1.44% (upper tertile) after 6 months of treatment had a lower cumulative MACE incidence compared with participants with GLS absolute increase <1.44% (log rank *P* = 0.015) (*Figure 2*). In detail, only 1 out of 99 patients with GLS absolute increase of ≥1.44% had a MACE (absolute risk 1.01%), whereas 25 out of 199 with a GLS absolute increase of <1.44% had a MACE (absolute risk 12.5%).

Patients with MACE had an impaired DT, *E*, *E*/*E*ʹ, and LA volume index compared with those without MACE (*P* < 0.05, *Table 2*). Conversely, *E*, IVRT, *S*ʹ, and RV *S*ʹ were similar between the two study groups (*P* > 0.05). By univariate analysis, DT, *E*ʹ, *E*/*E*ʹ, and LA volume index were predictors of adverse outcome (*P* < 0.05, *Table 3*). However, in multivariable analysis including SCORE2, diabetes, PASI, LVEF, PWV, treatment with ACEis/ARBs, lipid-lowering drugs, and using a backward procedure, none of the above markers showed an additive value to SCORE2 for the prediction of MACE (data not available, *P* > 0.05).

## Discussion

In the present study, we have demonstrated that GLS has a predictive value for MACE in psoriasis patients during a 4-year follow-up period. Baseline GLS, as well as GLS absolute change after 6 months of treatment with biological agents, had an independent and additive predictive value for MACE to SCORE2. A cut-off value of 16.4% for baseline GLS (lower tertile) and GLS absolute increase after treatment with a cut-off value of a GLS change ≥1.44% (upper tertile) were indicative of higher MACE-free survival during the follow-up period.

To our knowledge, this is the first study to assess the prognostic value of GLS and PWV for MACE in psoriasis patients. Psoriasis is characterized by a higher incidence of MACE compared with the general population.^18^ It has been demonstrated that incidences of myocardial infarction per 1000 person-years were 4.04 (95% CI: 3.88–4.21) for patients with mild and 5.13 (95% CI: 4.22–6.17) for patients with severe psoriasis.^6^ Results from a meta-analysis have indicated an elevated risk of CV events in psoriatic patients compared with controls without psoriasis [odds ratio (OR) 1.28; 95% CI: 1.18–1.38].^19^ The association between psoriasis and CV disease seems to be multifactorial. The increased prevalence of CV risk factors such as diabetes, hyperlipidaemia, and hypertension may partially explain the accelerated atherosclerosis in psoriasis patients.^12,20^ Psoriasis patients are at an increased risk for developing hypertension, diabetes, hypertriglyceridaemia, and quantitative and qualitative alterations in LDL and HDL cholesterol, which have been linked to accelerated atherosclerosis in these patients.^20-22^

Indeed, in our study, we observed that the presence of diabetes, hyperlipidaemia, hypertension, and a validated score to assess CV risk are univariate predictors of MACE at follow-up. However, most of the above risk factors have been shown to exert a detrimental effect on myocardial deformation even in the presence of normal LVEF as well as on arterial elasticity.^23^

Another important factor of the atherosclerotic process in psoriasis is systemic inflammation affecting CV function independently from the presence of CV risk factors. It has been demonstrated that psoriasis is associated with an increased risk for CAD regardless of age and the presence of diabetes, hypertension, or smoking.^24^ Moreover, psoriatic patients, particularly those with severe disease, have an increased risk of stroke and CV mortality regardless of the presence of traditional CV risk factors.^25,26^ Similarly, low-grade inflammation has been shown to adversely affect myocardial deformation and vascular function at an early stage.^5^ Indeed, in our study, we observed an association between impaired myocardial deformation as assessed by GLS and psoriatic disease duration. This finding suggests that exposure to an inflammatory state for a long period may directly compromise myocardial function and thus affect prognosis. There is growing evidence regarding the link between psoriasis and atherosclerosis, including the presence of activated T helper1 and T helper17 lymphocytes, macrophages, and monocytes in both psoriatic and atherosclerotic plaques. The inflammatory cytokines released in psoriatic lesions, including interleukin-6, 12, 17, 23, and TNF-α, are also implicated in myocardial and vascular dysfunction and atherosclerotic plaque formation and destabilization.^27-29^

Several studies have described early abnormalities of myocardial and vascular function in psoriasis related to disease duration, oxidative stress, and inflammatory markers.^5,30,31^ Therefore, markers of myocardial deformation and arterial stiffness including GLS and PWV, respectively, may identify subtle abnormalities of CV function attributed to an elevated inflammatory burden and/or the presence of atherosclerotic risk factors at an early phase of the disease and despite a normal LVEF.

Interestingly, treatment with biological agents resulted in a significant improvement of GLS and PWV in parallel with a reduction in disease severity, inflammatory, and oxidative stress markers.^32,33^ Indeed, previous studies have shown that the reduction of malondialdehyde, a marker of oxidative stress, and inflammatory cytokines, namely interleukin-12 and 6, after treatment with biological agents were associated with a concomitant improvement of PWV and GLS in psoriasis.^32,33^ Furthermore, in the above studies, improved PWV after treatment was also related to an increase of GLS. These findings support that reduction of oxidative stress and inflammatory burden may improve LV performance either directly and/or through improvement of arterial elasticity. Circulating cytokines including interleukin-6 and tumour necrosis factor TNF-α, and oxidative stress may have negative inotropic effects, result in apoptosis, and LV dysfunction.^34,35^ Increased arterial stiffness leading to early systolic arrival of wave reflections promotes a dysfunction of the subendocardial fibres of LV. Dysfunction of the subendocardial layer is a major determinant of impaired longitudinal myocardial deformation.^36^ Conversely, improved arterial elasticity permits arrival of wave reflection at diastole instead of systole and thus increases diastolic perfusion pressure to coronary arteries and reduces LV afterload.^36^ Thus, improved arterial elasticity after treatment with biological agents may improve longitudinal myocardial function by reducing afterload and increasing myocardial perfusion in psoriatic patients.

Global longitudinal strain is the most validated and widely applied parameter of myocardial deformation and is considered a useful and clinically applicable marker of subclinical myocardial dysfunction.^37^ A cut-off value of GLS ≤ −20% is expected in healthy subjects.^37^ Global longitudinal strain has an additive prognostic value to LVEF after myocardial infarction.^38^ Impaired GLS values have also been reported in psoriatic arthritis^39^ and rheumatoid arthritis.^40^ Abnormal GLS is considered to reflect the results of systemic inflammation, fibrosis, and coronary microcirculatory dysfunction on myocardial function in autoimmune rheumatic diseases (ARDs).^5,41^ Since cardiac involvement in psoriasis is a serious complication likely leading to increased morbidity and mortality,^3,6-8^ early detection of CV abnormalities is crucial to permit appropriate therapy and intensive modification of CV risk factors to reduce CV risk. The role of imaging parameters for the risk stratification for adverse cardiac events in ARDs remains unclear. It has been indicated that impaired myocardial deformation is associated with poor clinical outcomes in rheumatoid arthritis patients.^42^ In concordance with these data, we have demonstrated in the current study that GLS may a serve as an independent and additive prognostic marker to SCORE2 for CV events in psoriasis patients, as it may reflect the cumulative effects of an increased inflammatory burden and coexisting comorbidities on cardiac function at an early phase of the disease.

Interestingly, in our study, patients free of MACE had a greater improvement of GLS values after treatment with biological agents compared with those who developed MACE, suggesting that the beneficial effects of anti-inflammatory treatment on myocardial deformation may be translated to improved prognosis in patients with moderate to severe psoriasis. Targeting inflammation may have beneficial effects in terms of MACE reduction, as has been recently shown by the Canakinumab Anti-inflammatory Thrombosis Outcomes Study (CANTOS) trial.^43^ Pulse wave velocity has been also proposed as a parameter that may re-stratify patients to a higher CV risk beyond established CV factors.^44^ In our study, we found that PWV was also a univariate predictor of MACE in psoriasis patients. However, PWV did not maintain its predictive value in our multivariable model, possibly due to the co-existence of diabetes and hypertension, which are known to significantly affect arterial stiffness.^44^ However, in our study, improved PWV values after treatment with biological agents were observed only in patients free of MACE but not in those with MACE, suggesting that improvement of arterial function may have also contributed to improved cardiac performance, as previously shown,^32,33^ and consequently, to improved outcome in the long term. Echocardiography markers of LV diastolic function were univariate predictors of outcome in our study. Similar to PWV, these markers did not show an additive predictive value to SCORE2 by multivariable analysis, likely because of the co-existence of diabetes and hypertension, which are known to significantly affect LV diastolic function.

### Study limitations

This study is a single-centre study, with a relatively small size study population and a relatively low prevalence of MACE; thus, a limited number of covariates may have been included in multivariable analysis. The lower and non-statistically significant improvement of PWV compared with the respective statistically significant improvement of GLS after treatment in patients with MACE might be attributed to the much lower power of the test in the small sample size of patients with MACE.

Non-invasive imaging techniques such as stress echocardiography and coronary CT angiography were used to exclude significant obstructive coronary artery disease but may not rule out non-obstructive atherosclerosis. Thus, it is difficult to assess the impact of intermediate coronary artery stenosis on GLS values and increased risk of CV events. This study design does not permit us to fully elucidate the causality between baseline GLS values and its changes after treatment with MACE. Our study includes patients with moderate to severe psoriasis, and thus, the findings may not be extrapolated to psoriatic patients with mild disease, where the prevalence of traditional risk factors and calculation of SCORE2 may be the most important factors to assess CV risk and thus define proper treatment (e.g. statins) to alter prognosis.

In our study population, incidence of hypertension, diabetes, hyperlipidaemia, and smoking was relatively high, limiting the number of patients without any CV risk factors. Thus, an assessment of the prognostic value of GLS in psoriatic patients without risk factors was not feasible. As the presence of CV risk factors is common in psoriatic patients in the real world, the additive prognostic value of GLS assessment to that of SCORE2 in our study enhances the clinical utility of this echocardiography marker.

## Conclusions

Impaired baseline GLS and GLS change after treatment with biological agents had an independent and additive prognostic value to SCORE2 for MACE during a 4-year follow-up in psoriasis, after adjusting for several confounders and medication. Among all echocardiographic parameters in our study, GLS was the only one to show additive predictive value to SCORE2. Therefore, GLS may serve as a risk stratification marker, which enables taking a timely decision for providing an intensive anti-inflammatory treatment and aggressively modify CV risk factors to reduce CV risk in psoriasis patients.

## Data Availability

The authors state that data are avilable on reasonable demand.

## Lead author biography

Dr Ignatios Ikonomidis, MD, PhD, is Professor of Cardiology, Director of Echocardiography, Laboratory of Preventive Cardiology Clinic of Cardiometabolic Diseases, 2nd Cardiology Department, National and Kapodistrian University of Athens (NKUA), Attikon Hospital, Athens, Greece. In 1997, he completed his training in Cardiology and cardiac imaging, in Hammersmith Hospital, Imperial College, London, UK. Since then, he has been working as a consultant cardiologist in University Hospital of Patras, Department of Clinical Therapeutics and 2nd Cardiology Department, Medical School, Athens. His research fields include cardiovascular imaging, microcirculation, inflammation, diabetes, smoking control, and ventricular–arterial coupling. He authored 300 publications in indexed journals and several book chapters.
